# Multi-factorial considerations for intra-thoracic lymph node evaluations of healthy cats on computed tomographic images

**DOI:** 10.1186/s12917-021-02771-7

**Published:** 2021-01-28

**Authors:** Ninlawan Thammasiri, Chutimon Thanaboonnipat, Nan Choisunirachon, Damri Darawiroj

**Affiliations:** 1 Department of Surgery, Faculty of Veterinary Science, Chulalongkorn University, 39 Henri-Dunant Road, Wangmai, Pathumwan, Bangkok, 10330 Thailand; 2 Department of Anatomy, Faculty of Veterinary Science, Chulalongkorn University, 39 Henri-Dunant Road, Wangmai, Pathumwan, Bangkok, 10330 Thailand

**Keywords:** Cat, Computed tomography, Lymph node, Slice thickness, Thorax, Thymus

## Abstract

**Background:**

It is difficult to examine mild to moderate feline intra-thoracic lymphadenopathy via and thoracic radiography. Despite previous information from computed tomographic (CT) images of intra-thoracic lymph nodes, some factors from animals and CT setting were less elucidated. Therefore, this study aimed to investigate the effect of internal factors from animals and external factors from the CT procedure on the feasibility to detect the intra-thoracic lymph nodes. Twenty-four, client-owned, clinically healthy cats were categorized into three groups according to age. They underwent pre- and post-contrast enhanced CT for whole thorax followed by inter-group evaluation and comparison of sternal, cranial mediastinal, and tracheobronchial lymph nodes.

**Results:**

Post contrast-enhanced CT appearances revealed that intra-thoracic lymph nodes of kittens were invisible, whereas the sternal, cranial mediastinal, and tracheobronchial nodes of cats aged over 7 months old were detected (6/24, 9/24 and 7/24, respectively). Maximum width of these lymph nodes were 3.93 ± 0.74 mm, 4.02 ± 0.65 mm, and 3.51 ± 0.62 mm, respectively. By age, lymph node sizes of these cats were not significantly different. Transverse lymph node width of males was larger than that of females (*P* = 0.0425). Besides, the detection score of lymph nodes was affected by slice thickness (*P* < 0.01) and lymph node width (*P* = 0.0049). Furthermore, an irregular, soft tissue structure, possibly the thymus, was detected in all juvenile cats and three mature cats.

**Conclusions:**

Despite additional information on intra-thoracic lymph nodes in CT images, which can be used to investigate lymphatic-related abnormalities, age, sex, and slice thickness of CT images must be also considered.

## Background

The lymphatic system includes the circulating lymph and lymphatic vessels and organs [1]. The lymph node is an organ located along the lymphatic vessels inside body cavities or scattered at various locations throughout the body. Generally, in the thoracic cavity of cats, lymph nodes are scattered at several locations, such as the dorsal thoracic, ventral thoracic, cranial mediastinal, and bronchial areas [2, 3]. However, only sternal, cranial mediastinal, and tracheobronchial lymph nodes are reported to be more easily detected clinically [4]. These lymph nodes can be enlarged due to several etiologies including inflammation, tumors, or viral infections such as feline immunodeficiency virus (FIV) [5], feline leukemia virus (FeLV) [6, 7], and feline infectious peritonitis (FIP) [8]. It has been reported that FIV-infected cats can be affected by generalized lymphadenopathy and this can remain for 2–9 months after infection [9]. In addition, metastatic intra-thoracic lymphadenopathy, especially at sternal lymph node is also well known in cats that are affected with thoracic mammary gland tumors [10]. Unlike surface lymph nodes including mandibular, pre-scapular, inguinal, or popliteal lymph nodes that can undergo primarily clinical evaluation by palpation so that information regarding their appearances, sizes, shapes, and consistencies during pathological conditions can be obtained [11], the evaluation of mild-to-moderate alterations of intra-thoracic lymph node is challenging. Despite several diagnostic methods for lymph nodes including radiography, lymphangiography and ultrasonography, their feasibility to detect intra-thoracic lymphadenopathy is limited.

Currently, computed tomography (CT) can provide more information of intra-cavity lymph nodes due to the volumetric imaging information. CT provides multi-planar images with computerized multi-gray scale displays that can enhance the sensitivity of organ detection. However, several factors need to be considered to evaluate lymph nodes. To illustrate, the surrounding nodal fat has been reported to be one of the factors influencing the visibility of lymph nodes because fat can enhance the ability to distinguish structural outlines between lymph nodes and enclosed soft tissue structures [12]. Furthermore, age is counted as an additional factor affecting the size of lymph node [13, 14]. It has been reported that age is negatively correlated with medial retropharyngeal lymph node volume [13] and abdominal lymph node length in cats [14]. Although much information of CT appearances of feline lymph nodes was stated [13, 14], only retrospective information about feline intra-thoracic lymph nodes is available [4]. It has been reported that intra-thoracic lymph nodes of cats were not affected by age, sex and body weight (BW) [4]. However, that report was investigated in cats whose age ranged from 1.75 to 21.00 years old, in which the youngest cat was in the junior stage of the feline life stage [15]. Besides, several factors, such as gonadal status, body condition score (BCS) and the CT slice thickness that might affect the feasibility to detect lymph nodes, have never been reported. As a result, this study hypothesized that 1) younger cats such as kittens might have a larger intra-thoracic lymph node than those of felines at other stages of life and 2) gonadal status, BCS and CT slice thickness can affect the feasibility of detection of intra-thoracic lymph nodes in cats by means of lymph nodes size and detection score. Therefore, this study aimed to investigate the effect of both internal factors from the animals such as age, sex, gonadal status, BW and BCS and external factors such as the CT-associated setting and contrast enhancement on feline intra-thoracic lymph node appearances and its perceptibility using a prospective study model in healthy cats.

## Results

**Fig. 1:**
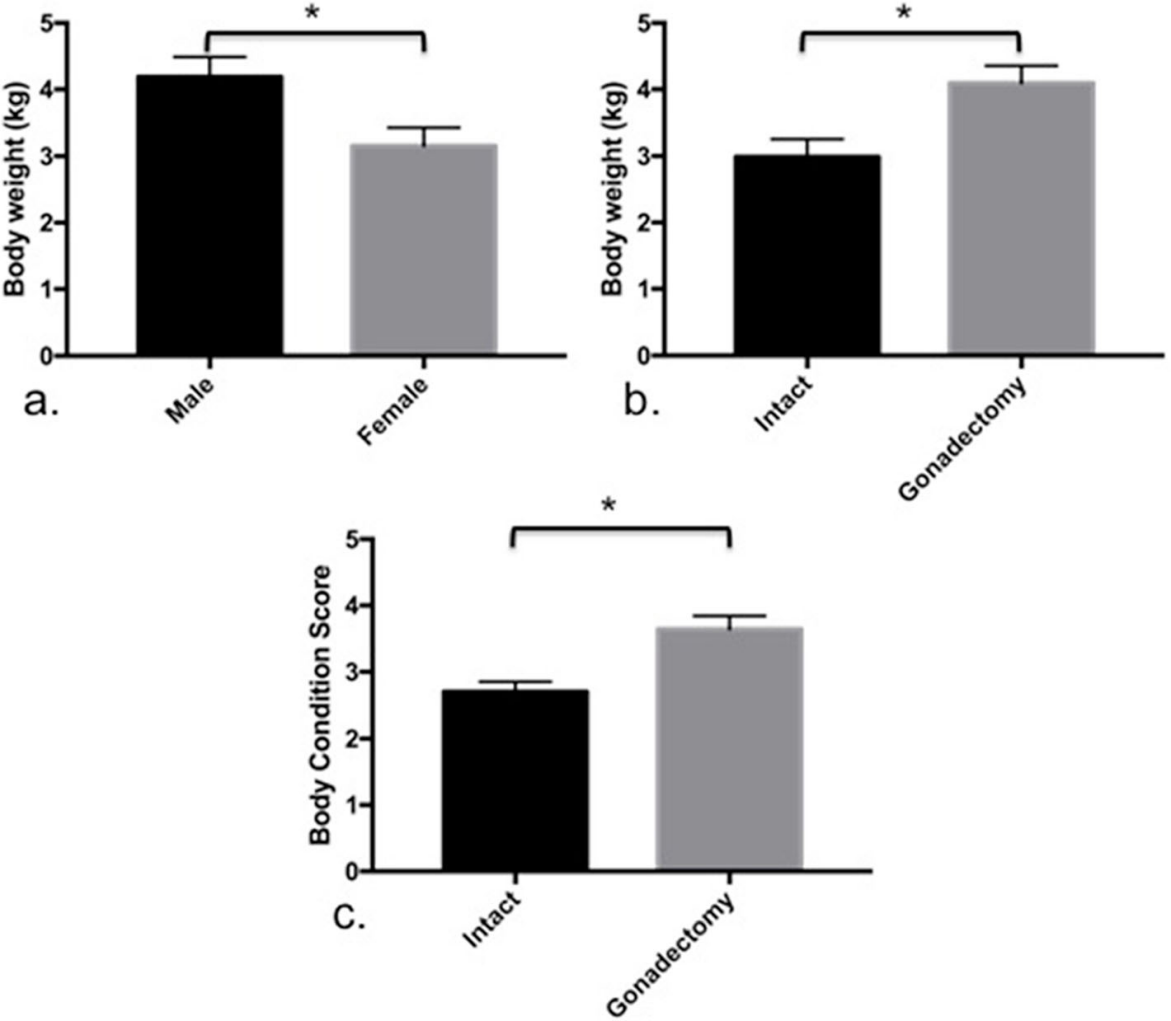
The comparative effect of gender (a) and gonadal status (b) on the body weight and the effect of gonadal status on body condition score (c) (asterisk: significant differences were detected)

During February and June 2017, twenty-four cats (8 cats in each age group) were included in this study. All cats have been clinically confirmed to be healthy and did not positive to any endemic viral diseases that may relate to the pathology of lymph nodes. The average age and BW of the study population was approximately 3 years and 8 months and 3.63 ± 0.21 kg, respectively. The most common breed of cats in the study was domestic short hair (*n* = 15), followed by mixed breed (*n* = 4), Scottish fold (*n* = 2), and a cat from American short hair and Persia. There were 14 male and 10 female cats. By gonadal status, there were 10 intact cats (6 intact females and 4 intact males) and 14 gonadectomized cats (8 spayed females and 6 castrated cats). The male cats had a significantly greater BW (*P* = 0.0228, Fig. 1a), but this difference was not detected by BCS (*P* = 0.1106). The gonadectomized cats had a significantly higher BW and BSC than the intact cats (*P* = 0.0092 and *P* = 0.0013, respectively; Fig. 1b and c). Information regarding the gonadal status, BW, and the BCS of each age group is presented in Table 1.

**Table 1:** Clinical demographic data including the mean and standard error of mean among twenty-four healthy cats

Clinical featuress		Total	Group1	Group2	Group3
Number of cats		24	8	8	8
Age (months)					
Average		44.58 ± 9.19	6.25 ± 10.41^£^	24.00 ± 4.53	103.50 ± 6.77^£^
Median		24.00	7.00	24.00	96.00
Range		4.00–132.00	4.00–7.00	12.00–48.00	84.00–132.00
Sex	**Female**	14	5	4	5
	Intact	6	4	2	0
	Spayed	8	1	2	5
	**Male**	10	3	4	3
	Intact	4	3	1	0
	Castrated	6	0	3	3
Weight (kg.)					
Average		3.63 ± 0.21	2.90 ± 0.30^α^	3.75 ± 0.36	4.25 ± 0.33^α^
Median		3.63	2.90	3.75	4.25
Range		1.50–5.70	1.50–4.70	2.00–5.00	2.90–5.70
Body condition score					
Average		3.25 ± 0.16	2.62 ± 0.18^β^	3.25 ± 0.25	3.87 ± 0.22^β^
Median		3.00	3.00	3.00	4.00
Range		2.50–5.00	2.00–3.00	2.00–4.00	3.00–5.00

Statistically difference among groups was made using Kruskal-Wallis test, α: *P* < 0.05; β: *P* < 0.01; £: *P* < 0.0001

Group1: cats aged ≤7 months; Group 2: cats aged > 7 months – 7 years; Group 3: cats aged ≥7 years

### The appearances of intrathoracic lymph nodes

**Fig. 2:**
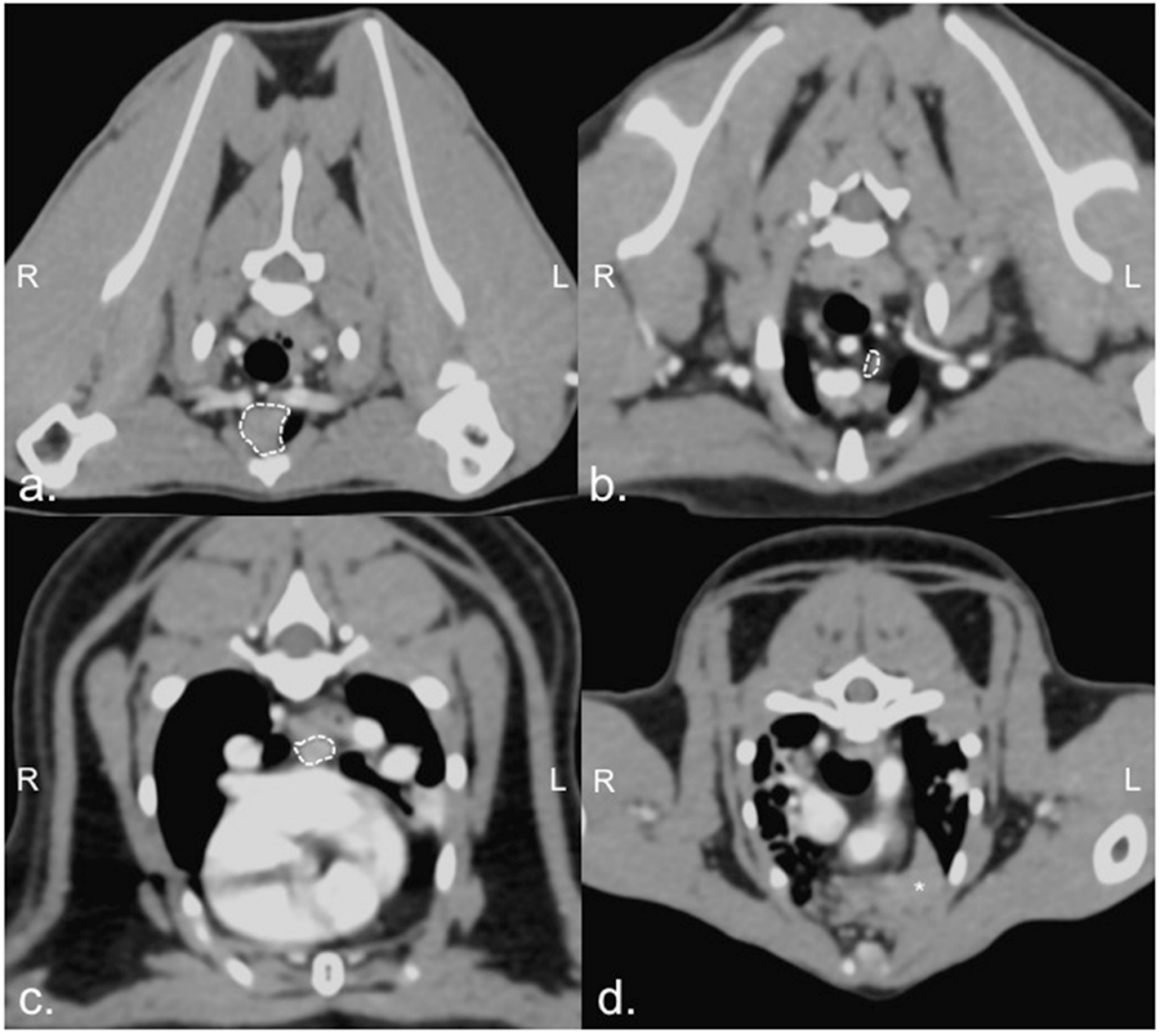
The soft tissue window (window width: 350 Hounsfield Unit (HU) and window level: 40 HU), transverse computed tomographic post-contrast image of feline thorax indicated the sternal lymph node (white dash line; a) the cranial mediastinum lymph node (white dash line; b), the tracheobronchial lymph node (white dash line; c), and suspected thymus (asterisk; d)

Thoracic CT images of all selected cats revealed no intra-thoracic abnormalities of pulmonary parenchyma and other related structures. For intra-thoracic lymph nodes, it was difficult to identify all intra-thoracic lymph nodes on the pre-contrast enhanced CT images. However, after intravenous contrast administration, CT images demonstrated sternal (6/24), cranial mediastinal (9/24), and tracheobronchial lymph nodes (7/24). The shape of all lymph nodes was elliptical with homogeneous, contrast-enhanced, soft tissue parenchyma. The attenuation number of detected lymph node on post-contrast enhanced CT image was 90.19 ± 7.30 Hounsfield Unit (HU) (median = 100.39 HU, range: 15.75–143.40 HU). Fat nodal hilus was detected in 7 lymph nodes from 6 cats (score 1 = 4 lymph nodes and score 2 = 3 lymph nodes). Fat at nodal hilus was detected in 3 cats each of G2 and G3, whose BCS ranged between 3 and 5 (median = 4). Consideration their locations, the sternal lymph nodes were embedded in fat above the second sternebra (Fig. 2a), while cranial mediastinal lymph nodes were scattered (at least 1–2 nodes) in the cranial mediastinal fat next to the large vasculatures (Fig. 2b). The tracheobronchial lymph nodes were normally found (at least 1–4 nodes) around the tracheal bifurcation (Fig. 2c). Inter-age group comparisons revealed that the lymph nodes were clearly visualized only in G2 and G3. The number of presented lymph nodes at each location or groups including the average transverse width and dimension are reported in Table 2. The dimension of lymph nodes at each location was not significantly different between G2 and G3. In addition to an inability to detect any lymph nodes in G1, a large, irregular soft tissue structure at the left cranial hemithorax was detected in all cats of G1 and some cats (3/8) of G2 (Fig. 2d).

**Table 2:** The number of the cats, number of the lymph nodes and the dimension of the lymph nodes (mm) such as width, length, height among intra-thoracic lymph nodes of sternal, cranial mediastinal, and tracheobronchial on post-contrast enhanced computed tomographic images

Location	Parameters	Total	Group 1	Group 2	Group 3
**Sternal lymph node**	Number of cats	6/24	–	3/8	3/8
	Number of lymph nodes	6	–	3	3
	Dimension of lymph node	3.93 × 8.39 × 3.84	–	4.46 × 9.75 × 4.60	3.41 × 7.02 × 3.09
**Cranial mediastinal lymph nodes**	Number of cats	9/24	–	2/8	7/8
	Number of lymph nodes	12	–	3	9
	Dimension of lymph node	4.02 × 6.23 × 3.67	–	2.97 × 6.52 × 3.14	4.38 × 6.17 × 3.98
**Tracheobronchial lymph nodes**	Number of cats	7/24	–	2/8	5/8
	Number of lymph nodes	10	–	2	8
	Dimension of lymph node	3.51 × 4.93 × 3.44	–	2.67 × 2.92 × 2.36	3.73 × 6.81 × 3.54

Group1: cats aged ≤7 months; Group 2: cats aged > 7 months – 7 years; Group 3: cats aged ≥7 years

Furthermore, among the 14 cats that clearly demonstrated the intra-thoracic lymph nodes, the males had a larger transverse overall lymph node width than that of the females (*P* = 0.0425). In contrast, BW and BCS were not correlated to lymph node width. In addition, dividing by the locations of lymph nodes, transverse lymph node width did not statistically significant difference between factors of sex, BW and BCS (Table 3).

**Table 3:** Factors that affected to the size of intra-thoracic lymph nodes on computed tomographic images

Factors		Lymph node width (mm)	Difference ***P***
Age	≤ 7 months		0.5536
		–	–
	≥7 months – 7 years	3.39 ± 0.53	
	≥7 years	4.03 ± 0.50	
Sex	Overall lymph nodes		0.0425^*^
	Male	4.41 ± 0.63	
	Female	3.31 ± 0.43	
	Sternal lymph node		0.7000
	Male	4.56 ± 0.82	
	Female	3.30 ± 1.29	
	Cranial mediastinal lymph node		0.2546
	Male	5.13 ± 1.49	
	Female	3.47 ± 0.63	
	Tracheobronchial lymph node		0.4762
	Male	3.87 ± 0.96	
	Female	2.99 ± 1.57	
Gonadal status			0.5806
	Intact	4.33 ± 1.03	
	Gonadectomy	3.76 ± 0.41	
Body weight	Overall;	3.82 ± 0.38	0.2344
	Range: 1.50–5.70 kg		
	Sternal lymph node;	3.93 ± 0.74	0.7632
	Range: 3.10–5.70 kg		
	Cranial mediastinal lymph node;	4.02 ± 0.65	0.2692
	Range: 2.80–5.70 kg		
	Tracheobronchial lymph node;	3.51 ± 0.62	0.2684
	Range: 2.00–5.70 kg		
Body condition score	Overall;	3.82 ± 0.38	0.1841
	Range: 2.0–5.0		
	Sternal lymph node:	3.93 ± 0.74	0.3138
	Range: 3.0–5.0		
	Cranial mediastinal lymph node;	4.02 ± 0.65	0.0755
	Range: 3.0–5.0		
	Tracheobronchial lymph node;	3.51 ± 0.62	0.2998
	Range: 2.0–5.0		

^*^The statistical different between groups was made using Mann-Whitney test

The correlations between Lymph node width and Body weight, Body condition score or Degree of obesity were made using Spearman correlation

### The CT slice thickness for intra-thoracic lymph nodes

The post-contrast-enhanced CT data could only be identified and scored for 14 cats. Slice thickness at 0.625 mm provided the highest detection score of the intra-thoracic lymph nodes, followed by the slice thicknesses at 1.250, 2.500, and 5.000 mm, respectively. There was no statistically significant difference in the detection score from slice thicknesses between 0.625 mm and 1.250 mm. In comparison to other slice thicknesses, the detection score from each of slice thickness at 0.625 or 1.250 mm were significantly different to those at 2.500 or 5.000 mm (Table 4).

**Table 4:** Factors that affected to the detection of intra-thoracic lymph nodes detection on computed tomographic images

Factor		Detection score	Difference ***P***
Slice thickness (mm)	0.625 vs 1.250	1.68 ± 0.10 vs 1.45 ± 0.12	> 0.9999
	0.625 vs 2.500	1.68 ± 0.10 vs 0.72 ± 0.09	0.0003^*^
	0.625 vs 5.000	1.68 ± 0.10 vs 0.13 ± 0.07	< 0.0001^*^
	1.250 vs 2.500	1.45 ± 0.12 vs 0.72 ± 0.09	0.0109^*^
	1.250 vs 5.000	1.45 ± 0.09 vs 0.13 ± 0.07	< 0.0001^*^
	2.500 vs 5.000	0.72 ± 0.09 vs 0.13 ± 0.07	**0.0675**
Sex	Male	1.77 ± 0.14	
	Female	1.61 ± 0.14	
Gonadal status	Intact	2.00	
	Gonadectomy	1.65 ± 0.10	
Lymph node width (mm)	Range: 1.72–9.54	1.68 ± 0.10	0.0049^α^
Body weight (kg)	Range: 1.50–5.70	1.68 ± 0.10	0.1327
Body condition score	Range: 2.50–5.00	1.68 ± 0.10	0.3180

*The statistical different among slice thicknesses was made using Kruskal–Wallis test

The statistical different between male and female was made using Mann-Whitney test

^α^The correlations between Detection score and Lymph node width, Body weight, Body condition score or Degree of obesity were made using Spearman correlation

In addition to the variations in the slice thickness, the detection score was not correlated to BW or BCS after controlling the slice thickness at 0.625 mm. However, the lymph node size demonstrated an effect on the detection score (*P* = 0.0049, r = 0.5768) (Table 4).

## Discussion

Intra-thoracic lymph nodes could be clearly detected only on post-contrast enhanced CT images. Without enhancement of contrast medium, the inability to distinguish between lymph nodes and surrounding soft tissues in the feline thorax could be accounted as a limitation. Therefore, the contrast-enhanced technique could be applied to the CT procedure to ensure a greater degree of precision in the images. Although use of an intravenous contrast medium was routinely followed in CT scan to improve image quality and facilitate the differentiation between normal and pathological tissue [16], all intra-thoracic lymph nodes remained undetectable in kittens. This might be due to several reasons, such as the smaller size of the lymph node, lesser fat accumulation in the mediastinum or adjacent structures that affects the evidence of lymph node boundary, and the presence of an enlarged, thymus-suspected soft tissue structure that might later obscure the smaller lymph nodes. In dogs, progressive involution of thymus could be found between the ages of 6 and 23 months [17]. However, the degeneration time of the thymus in cats had not been reported. Considering this, the oldest cat presenting with a thymus-suspected structure was aged 12 months. However, this was not conclusive and further studies are needed.

BW and BCS were described as factors that effected to a feasibility of lymph node detection in the previous studies [12, 13]. In addition to the higher BW in male cats, the BW and BCS were significantly greater in gonadectomized cats than those of both intact genders. The results of this study were similar to that of a previously published investigation [18]. The gained BW in gonadectomized cats was caused by hormonal variations, which subsequently increased food intake [19–21]. The greatest average BW and BCS, specifically in senile cats, was assumed to affect the feasibility of detecting intra-thoracic lymph nodes considering the clear fat-lymph node demarcation. In dogs, the surrounding fat formed a well-defined boundary to the lymph node [12]. However, the statistical differences could not be visualized when comparing the feasibility to detect lymph nodes and the factors associated with BW or BCS in this study. In contrast, the feasibility of detection was significantly correlated to the size of the lymph nodes. Therefore, a larger lymph node size was associated with an ease of detection, which was consistent with the outcomes of a previous report [12].

Juvenile and mature cats revealed fewer lymph nodes on the CT images. With regard to the BCS, senile cats typically presented with higher BC and BCS. Besides, the larger dimensions of lymph nodes, especially at the cranial mediastinal and tracheobronchial areas, were detected in this group. This could be attributed to the fat accumulation at the hilus of the lymph node [13]. Loss of the evidence of nodal hilus on diagnostics imaging was associated with neoplastic infiltration in multiple species [22, 23]. However, the disappearance of the fat accumulation at the nodal hilus in present study might be due to the small size of most lymph nodes. Currently, there are no studies that have reported the relationship between hilus of lymph node and dimensions of the lymph node. Therefore, it was assumed that a higher BCS in cats was associated with a greater degree of fat deposition at the hilar region of lymph nodes as seen in some cats of this study. However, the relationship between either BCS or the accumulation of fat at the hilus and a dimension of lymph nodes should be further investigated.

The maximal width of lymph node on transverse CT image, a primary axis of CT diagnosis in veterinary medicine, was utilized to statistically observe several aspects in present study. The mean ± SD of the widest of sternal lymph node, cranial mediastinal lymph node, and tracheobronchial lymph nodes were 3.93 ± 0.74 mm, 4.02 ± 0.65 mm, and 3.51 ± 0.62 mm, respectively. Previously, a report had quantitatively assessed the tracheobronchial and sternal lymph nodes after inoculation with *Aelurostrongylus abstrusus (A. abstrusus)* in 6 cats [24]. The results showed that the mean ± SD of pre-inoculated dimensions of intra-thoracic lymph nodes were similar to that of our results. Subsequently, after infection at 48 and 81 days, moderate lymphadenopathy due to the reactive hyperplasia was detected through thoracic radiographs and post-contrast enhanced CT image with an increased CT attenuation of the affected lymph nodes. Previous studies reported that CT attenuation of normal intra-thoracic lymph node at pre- and post-contrast enhancement were 7.3–60 and 8–263 HU, respectively [4, 14]. Whereas, the CT attenuation at post-contrast CT image were ranging form 15.75–143.40 HU. Therefore, the estimated attenuation number of intra-thoracic lymph nodes at pre- and post-contrast enhancement should be 7.3–60 and 8–263 HU. Recently, Smith et al. (2019) reported that the size of presumptively normal intra-thoracic lymph nodes was not affected by age, weight and sex [4]. However, that study was performed in adult cats whose ages began from 1.75 years. At that age, a similar result was observed, implying that lymph node size in G2 and G3 was not significantly different. A comparison between studies showed that the mean widths of sternal, cranial mediastinal, and tracheobronchial lymph nodes in the previous study were 3.0, 2.1 and 2.4 mm, respectively, which were smaller than our results. This might be due to the difference in age ranges between studies; the previous study was conducted in cats whose age range was between 1.75 and 21 years (median = 7.5 years), whereas in the present study, the cats’ age ranged from 4 months to 11 years (median = 2 years). The smaller size of lymph nodes in previous study might be due to age-related replacement of immune cell by connective tissue [25].

Although sex is not a factor on lymph node size, gonadectomized male cats have a longer lymph node than those of the spayed females in the previous study [4]. Considering the sex, male cats were significantly heavier than female cats. Therefore, body size might affect the lymph node size, both in the previous and the present study. Last but not least, present study was evaluated the effect of gonadectomy on the lymph node size. Although it has been reported that gonadectomy causes a decrease in androgens in post-pubertal male mice due to its effect on the lymphoid organs by increasing immune cells [26]; the different sizes of lymph nodes between intact and gonadectomized cats could not be detected in our study. The discrepancy between studies might be due to the inter-species difference or a smaller population size in the present study. Therefore, prospective study with a larger population and control protocol might be further required.

The resolution of the CT image is a crucial goal for diagnostic quality. Detectability of anatomical structures, especially low-contrast organs, is related to several factors such as radiation dose requiring a combination of tube current and tube voltage, slice thickness, and pitch [27–29]. Radiation dose is the main factor required for the creation of a qualified image due to quantum detection at the image detector [27]. To create a valuable diagnostic CT image, higher signal-to-noise ratio (SNR) is desirable to produce a greater image quality. To achieve a higher level of SNR, radiation dose must be increased to reduce image noise [30]. There are several factors contributing to image noise of a CT image. For example: a lower kVp setting causes a greater photoelectric effect [28, 31]. Despite this, it can increase the contrast of the image and enhance visualization [28]; this effect on the attenuation of a photon in the object tissue, called photon starvation effect, can increase noise [27].

It is well known that slice thickness in also counted as a factor of image quality that contributes to the noise of an image [32]. Slice thickness is one of the factors that can influence the interpretation precision on CT images [27, 30]. Generally, slice thickness should be as low as possible to reduce partial volume effect (PVE) that is the effect of a thicker slice causing the less perceptibility of the smaller anatomical structures [33]. Despite decreasing PVE, too thin a slice could increase the image noise [32]. To conquer that problem, an increased radiation dose with an increased tube current can be used to increase SNR [28], however, it would result in a higher dose delivery to the patient.

The previous report unveiled that 1.5–2.0% of cancers may be related to the CT radiation [34]; the scanning technique in clinical practice must be optimized to achieve the diagnostic task with a higher imaging quality and lower delivered radiation dose following the concept of as low as reasonable achievable (ALARA). In addition, the scan range should reportedly be as small as possible due to the related total radiation dose delivered [27]. It is well known that dose-length product (DLP) is the total radiation dose absorbed by the patient with the relationship to number of slices and the scanning length as: DLP = CTDI × T × N where CTDI: CT dose index, T: slice thickness and N: number of slices [35]. It has been reported that a tube current has a linear relationship with slice thickness. When the slice thickness is increased, the higher radiation dose that produces the adequate number of photons to produce the image must be increased to reduce noise and increase the SNR [32]. From present study, slice thickness at 0.625 mm provided the highest detection score of the normal, intra-thoracic lymph nodes. Similar result was reported in a previous study with human participants, which suggested that the interpretation of CT images of children required a smaller slice thickness than that of the adults [36] to conquer with PVE. However, at the similar setting of mA, thinner slices of CT images can increase noise [32] and degrade image quality. In dogs, the recommended CT slice interval to examine the tracheobronchial lymph node was 1.0–1.5 mm [37], which was consistent with the results of the present study. In the retrospective CT study on feline lymph node the slice thickness was setup only at 1.5 mm [4]. Since the significant difference between the feasibility to detect feline intra-thoracic lymph nodes at the slice thicknesses of 0.625 and 1.250 mm could not be identified, the appropriate slice thickness for CT examination to enhance detectability with higher SNR of feline thorax should be 1.250 mm. At this CT thickness, the radiation risk to animals is reduced considerably, while ensuring a good SNR of CT image that would be suitable for data management.

In addition to the slice thickness at 1.250 mm, the present study was conducted under the automatic exposure control (AEC). AEC is the tube current modulation at the longitudinal (z) axis and angular (x-y) axes that can decrease dose delivery to the patient. Besides, this study was performed at 0.969 of pitch. A higher pitch can decrease motion artifact [27] that can enhance image resolution. In addition to the detectability and size of the intra-thoracic lymph nodes of cats in this study, a variety of exposure settings on the low contrast detectability (LCD) and dose delivery should be further investigated to optimize scanning techniques for higher image quality along with ALARA concept.

Despite the variation of the age groups, the small sample size with a limited breed of cats could be considered as a major limitation of this study. Due to the regulation of animal use for scientific research, the appropriate number of cats, which were screened to be a good representative in according to the inclusion criteria, was agreed and designed. Therefore, at this sample size, presented information could be applied and enlighten for clinical usage. In addition, bronchoscopy and coproscopy were not performed in this study because no incidence of the lung parasite in the area of this study was reported. Besides, all of the enrolled cats were indoor cats, which regularly underwent health checks and were updated with all preventive medicine. Moreover, according to the policy of animal use, additional invasive procedures such as tissue biopsy or necropsy to validate the anatomical tissue type could not be done for both intra-thoracic lymph nodes and suspected thymus. Further prospective studies with a larger study population, along with the validation to examine the gross anatomy of the feline thorax would be necessary.

## Conclusions

In conclusion, to examine the feline intra-thoracic lymph nodes by CT scan, internal and external factors need to be concerned. Age influences on the feasibility of lymph node detection. Contrary to previous reports, detection of normal, intra-thoracic lymph nodes was difficult in kitten. Besides, a large, irregular shape, soft tissue structure at the left cranial thoracic cavity that is possibly the thymus may obscure the lymph node detection to the age of 12 months. Nonetheless, intra-thoracic lymph nodes are detected with greater clarity in the adult cats that have the larger lymph node width, specifically in the male cats whereas gonadal status, BW and BCS do not affect lymph node detection. Additionally, for CT scanning protocol of intra-thoracic lymph nodes, post-contrast enhancement procedure with a slice thickness of 1.250 mm is suggested.

## Methods

### General materials

This study was approved by The Institutional Animal Care and Use Committee of Chulalongkorn University (CU-IACUC), the approval number: 1631073. Cats that were recruited in this study were indoor healthy cats visiting for purposes either of annual health checkup or request for gonadectomy, and volunteer cats from staffs. Cats were included if they were in healthy condition, aged more than 4 months old with the complete history of vaccination. In brief, information such as age, sex, gonadal status, BW, and BCS [15] were collected and all cats were sent for physical examination, hematology, basic screening of serum biochemistry following the hospital protocol including creatinine, blood urea nitrogen, alkaline phosphatase, alanine aminotransferase, total protein and albumin, urinalysis, serologic examination and the serologic examination of endemic viral diseases such as FIV and FeLV (Witness®, Zoetis, France). Subsequently, right lateral and ventrodorsal projections for thoracic and abdominal radiography were performed using a direct digital radiography X-ray machine (ETL®, General Electric, Beijing, China). In addition, abdominal ultrasonography was performed using a 7.0 MHz, linear transducer (LogiqP6®, General Electric, Seoul, Korea). Radiographic and ultrasonographic screenings were performed by a Diplomate Thai Board Veterinary Surgery and radiologist (NC). If any cat revealed any of history such as the previous or present conditions related to lymphadenopathy or lymphoid hyperplasia e.g. previous vaccination within fourteen days or affected with any diseases before attending the study, abnormalities from the above examination or were in a condition that may affect the anesthetic procedures, they were excluded from the study. Subsequently, cats were categorized into three age groups following the AAFP-AAHA Feline Life Stage Guidelines [15] as follows: group 1 (age ≤ 7 months or kittens), group 2 (age > 7 months – 7 years including junior and prime) and group 3 (age ≥ 7 years; including mature senior and geriatric).

### CT scan imaging protocol

Food and water were withheld for 8 h before the CT procedures. Cats were induced under general anesthesia through the following protocol: premedication with 2 mg/kg tramadol hydrochloride (SC, Tramache®, India) and 0.03 mg/kg acepromazine maleate (IM, Combistress®, Belgium), and generalized anesthetic induction with 2–4 mg/kg propofol (IV, Lipuro®, Germany). After the intubation, cats were subsequently maintained under anesthesia with 2–5% isoflurane (AERRANE®, USA) with oxygen through the ventilator (SV-2000, SOARMED, Taiwan) without the breath-hold technique at a respiratory rate of 12 times/minute throughout the CT procedure. The duration since the induction of anesthesia until the scout phase of CT was done in all cats was approximately 10 min. Briefly, cats were placed in a sternal position and the thoracic cavity was perpendicular to the isocenter of CT gantry. Initially, the scout phase was conducted with a 64-slice, helical CT unit (Optima CT660, General Electric, Japan) at 120 kVp and 10 mA in all cats. Subsequently, the field of view (FOV) for both pre- and post-contrast CT images was created to cover all dimensions of the thoracic cage, ranging from thoracic inlet to the caudal area of the 13th thoracic vertebra. The pre-contrast enhanced CT images of the entire thorax were then collected using the following parameters: a low-pass filter, 1.25-mm slice thickness, 1.25–mm slice interval, collimator pitch at 0.969, matrix of 512 × 5.12, peak kilovoltage of 120 kVp, and automate amperage. Immediately, a 600 mg/kg iohexol contrast medium (Omnipaque 200®, Ireland) was manually administrated intravenously through the right cephalic vein and the post-contrast scanning was done within 30 s of contrast administration. During the acquiring of CT images, the window level (WL) was set at 30 HU and window width (WW) at 250 HU. The effect of CT slice thicknesses on a feasibility to detect intra-thoracic lymph nodes was evaluated by reconstructing the CT images to be 0.625, 1.250, 2.500, and 5.000 mm. All data were saved as Digital and Communication in Medicine (DICOM) files. After the CT procedure and full recovery from generalized anesthesia were achieved, all cats were allowed to return to the owner.

### The appearance of intra-thoracic lymph nodes on CT images

**Fig. 3:**
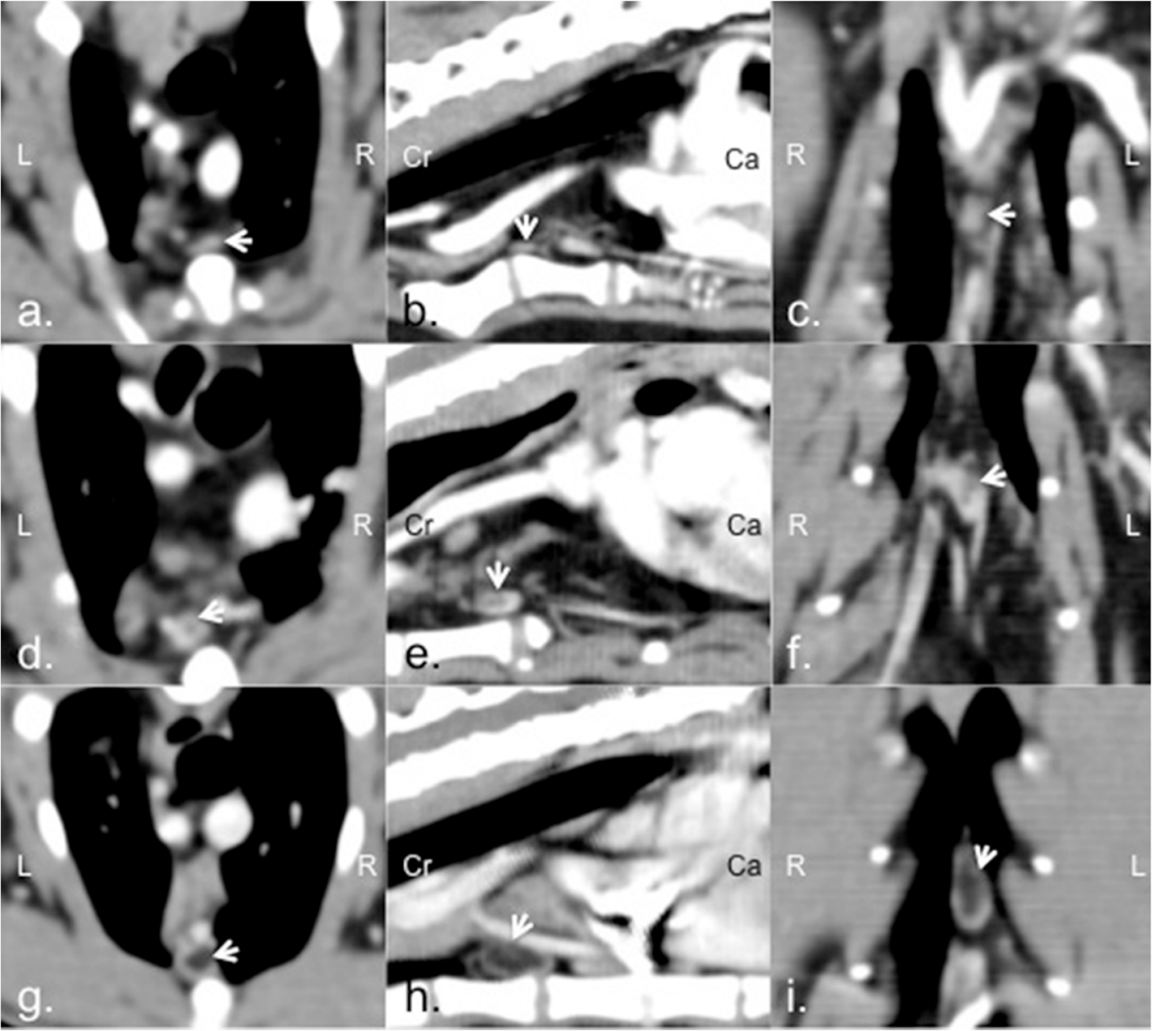
The presence of intra-thoracic lymph nodal hilus fat on each plane of transverse, sagittal or dorsal computed tomographic image. The nodal hilus fat was scored as 0, no evidence of nodal hilus fat (a, b, c); 1, evidence of nodal hilus fat but intranodal fat outline was not distinct (d, e, f); and 2, obvious evidence of nodal hilus fat with clear intranodal fat outline (g, h, i)

Prior the evaluation of intra-thoracic lymph node, all cats were evaluated for intra-thoracic abnormalities both pulmonary parenchyma and other related structures. Subsequently, multiplanar reconstruction was applied by a Diplomate Thai Board Veterinary Surgery and radiologist (NC) to enhance the evidence of intra-thoracic lymph nodes. A soft tissue window at 350 HU of WW and 40 HU of WL was set to ensure the best visualization in both intra-thoracic lymph nodes and the associated soft tissue structures. The evidence, number, and appearance of sternal, cranial mediastinal, and tracheobronchial lymph nodes on both pre- and post-contrast enhanced CT images were noted. The maximal dimensions of width, length, and height of the detected lymph nodes were measured and reported. In addition, the presence of nodal hilus fat was scored as 0, no evidence of nodal hilus fat; 1, evidence of nodal hilus fat but intranodal fat outline was not distinct; and 2, obvious evidence of nodal hilus fat with clear intranodal fat outline (Fig. 3).

### The effect of CT slice thicknesses on the detection of intra-thoracic lymph node

Slice thickness was a parameter of the CT machine that affected the feasibility of identifying intra-thoracic lymph nodes. Here, slice thicknesses at 0.625, 1.250, 2.500, and 5.000 mm of both pre- and post-contrast enhanced images were revealed and scored according to the following information: the feasibility of detection or a detection score was given 0 if the lymph node could not be detected, 1 if lymph node could be detected but the outline of the lymph node was not clear, and 2 if lymph node was detected with obvious appearance.

### Statistical analysis

All data were expressed as mean ± SEM. Prior to statistical analyses, Shapiro-Wilk test was applied to validate the normality distribution of all data. The differences of BW, and BCS among groups and the feasibility to detect lymph nodes among the various slice thicknesses were analyzed using Kruskal-Wallis test. The statistical differences of BW, and BCS, between sexes and gonadal statuses including the difference of transverse width of lymph node between groups or sexes were tested using Mann–Whitney U-test. The relationship between parameters such as BW, and BCS including transverse width of the lymph nodes and lymph node size or detection score were observed by Spearman rank test. All statistical analyses were performed using Prism7 (GraphPad software, USA), and a *P* value of less than 0.05 was considered as a statistically significant difference.

## Data Availability

The datasets used and/or analyzed during the current study are available from the corresponding author on reasonable request.

## Abbreviations

AEC: Automatic exposure control
ALARA: As low as reasonable achievable
BCS: Body condition score
BW: Body weight
CT: Computed tomography
CTDI: Computed tomographic dose index
CU-ACUC: Chulalongkorn University Animal Care and Use Committee
DICOM: Digital and communication in medicine
DLP: Dose-length-product
FeLV: Feline leukemia virus
FIP: Feline infectious peritonitis
FIV: Feline immunodeficiency virus
FOV: field of view
HU: Hounsfield unit
IM: Intramuscular injection
IV: Intravenous injection
Kg: Kilogram
kVp: Kilovoltage peak
LCD: Low contrast detectability
mA: Milliampere
mg: Milligram
mm: Millimeter
N: Number of slices
PVE: Partial volume effect
T: Slice thickness
SC: Subcutaneous injection
SEM: Standard error of mean
SNR: Signal-to-noise ratio
WL: Window level
WW: Window width
x-y axes: Angular axes
z axis: Longitudinal axis
